# In Vitro Evaluation of an Adhesive Hydrogel‐Based Chondrocyte Carrier for Enhanced Therapeutic Delivery

**DOI:** 10.1002/mabi.202500653

**Published:** 2026-04-09

**Authors:** Peyman Karami, Alexis Laurent, Virginie Philippe, Lee Ann Applegate, Dominique P. Pioletti, Robin Martin

**Affiliations:** ^1^ Department of Orthopedic Surgery and Traumatology Lausanne University Hospital University of Lausanne Lausanne Switzerland; ^2^ Laboratory of Biomechanical Orthopaedics, Institute of Bioengineering School of Engineering EPFL Lausanne Switzerland; ^3^ Manufacturing Department LAM Biotechnologies SA Epalinges Switzerland; ^4^ Regenerative Therapy Unit Reconstructive and Hand Surgery Service Lausanne University Hospital University of Lausanne Epalinges Switzerland; ^5^ Center For Applied Biotechnology and Molecular Medicine University of Zurich Zurich Switzerland; ^6^ Oxford OSCAR Suzhou Center Oxford University Suzhou China

**Keywords:** adhesive hydrogel, cartilage repair, human autologous chondrocytes

## Abstract

Large knee chondral defects remain a major challenge in orthopaedic surgery and may lead to osteoarthritis. Autologous chondrocyte implantation (ACI) is a widely used regenerative therapy; current techniques have inherent limitations related to cell delivery, retention, and integration. This study investigates a novel adhesive hydrogel as a cell carrier for enhanced therapeutic delivery in cartilage repair. This injectable hydrogel can lead to a paradigm shift in ACI through removing the need for membranes entirely and enabling direct in situ fixation of cell‐laden constructs while preserving the chondrocytic phenotype. In this work, we report in vitro encapsulation of human autologous chondrocytes (HACs) using cells from five orthopedic patients within this hydrogel with subsequent in vitro evaluation of functional outcomes, including cell viability, glycosaminoglycan production, histological and immunohistological outcomes, and evolution of mechanical properties of the cell constructs. Moreover, by analyzing the hydrogel polymer content (PC), we identified an optimal formulation balancing biological activity and mechanical integrity.

## Graphical abstract

An adhesive, light‐activated hydrogel is evaluated as a carrier for human autologous chondrocytes. The system supports high cell viability, extracellular matrix deposition, and progressive mechanical reinforcement in vitro. By optimizing polymer content, a formulation balancing biological activity and mechanical integrity is identified. This injectable platform shows promise for improving cell delivery and integration in cartilage repair applications.

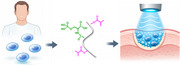

## 1 Introduction

Articular cartilage defects in the knee represent a major clinical challenge. These lesions may progress to osteoarthritis and ultimately necessitate joint replacement. Current treatment strategies in orthopedics provide only partial solutions, particularly in the case of defects where the self‐healing potential is limited.

Autologous chondrocyte implantation (ACI) has become one of the most well‐established biological approaches for cartilage repair promotion. Over the past three decades, it has undergone several iterations. Second‐generation ACI, which relies on synthetic or collagen membranes to secure the implanted cells, remains limited by problems of cell leakage, suboptimal integration within the lesion, and the need for open surgical procedures [1]. At the Lausanne University Hospital, we have extensive experience with this technique, as an extensively documented procedure with easier in‐house production rather than matrix‐induced procedures. Around 100 cases with the mean lesion size of > 4 cm^2^ have been treated since 2017, with clear improvements in patient scores and imaging follow‐ups [2]. However, we have identified room for therapeutic improvement, particularly in ensuring effective cell delivery and tissue integration. Third‐generation, or matrix‐induced ACI (MACI), is now widely adopted and has demonstrated less surgical morbidity, more uniform cell distribution, and low graft hypertrophy. However, comparative studies directly evaluating second‐ and third‐generation ACI are limited, with no proven superiority of MACI in clinical outcomes compared to second‐generation. In MACI, autologous chondrocytes are expanded and seeded onto a membrane scaffold under GMP conditions before implantation [3]. Despite the intended advantages, MACI still faces important limitations [4, 5, 6]: (a) the technical complexity of seeding HACs onto membranes requires specialized GMP facilities and experienced personnel; (b) the process needs a fixation method usually using fibrin glue which lacks suitable mechanical strength, therefore there is a risk of early delamination, and the incomplete integration; (c) adaptation to irregular defect geometries is imperfect and requires manual shaping; (d) persistent chondrocyte dedifferentiation and interindividual variability in chondrocyte redifferentiation contribute to heterogeneous clinical outcomes; and (e) chondrocytes are distributed in a 2D layer on the membrane surface. This limits their regenerative performance compared with 3D encapsulation and results in variable clinical outcomes.

To overcome these limitations, injectable ACI (fourth generation) has recently emerged [7, 8]. This approach is often characterized by injectable cell‐laden biomaterials that directly deliver chondrocytes, conform to the defect shape, and support matrix development. Although this generation represents a highly promising step forward, currently available systems (e.g., Novocart Inject, ChondroFiller, etc.) face important challenges, particularly in effective cell retention, delivery, and long‐term functionality. Thus, a clinical need for more advanced solutions remains [9, 10]. Indeed, an unmet need exists for an injectable cell carrier that combines strong adhesion and durable integration to native cartilage, and supports chondrocyte viability, phenotype, and high‐quality hyaline‐like matrix development over time.

Adhesive hydrogels could constitute promising solutions for cell delivery in fourth‐generation ACI [11]. With sufficient adhesion to surrounding tissue, they have the potential to provide secure integration to cartilage defects and mimic the native extracellular matrix (ECM) environment required for cartilage regeneration. However, current injectable hydrogels are mostly weak in tissue adhesion formation and lack the ability for robust matrix development. To address this need, we have previously developed a novel tissue‐adhesive hydrogel, MechaGel, which polymerizes rapidly upon blue light activation and adheres to cartilage [12]. In our previous proof‐of‐concept studies, we demonstrated the superior adhesion of our developed hydrogel to cartilage compared to existing clinical adhesives, as well as its in vivo performance in a large animal model [12, 13]. Using a goat cartilage defect model, we showed that the acellular hydrogel could provide lateral integration with surrounding cartilage after a six‐month follow‐up period.

Building on the promising development mentioned above briefly, the present study investigates our adhesive hydrogel as a cell carrier for human autologous chondrocytes. Specifically, we evaluate whether encapsulation within the hydrogel preserves cell viability, promotes glycosaminoglycan accumulation, supports the deposition of hyaline‐like extracellular matrix as assessed by histology and immunohistochemistry, and provides progressive mechanical reinforcement during culture. We also aim to evaluate the optimal formulation of the hydrogel platform in the context of its biomechanical and biological performance for the subsequent in vivo cell therapy investigations and future translational development.

## 2 Materials and Methods

### 2.1 Hydrogel Synthesis and Preparation

The synthesis of hydrogel pre‐polymer was described previously in our earlier studies [12, 13]. Gelatin (Type A, Sigma‐Aldrich, G2500) was used as the base polymer backbone, functionalized with methacrylate groups to enable photo‐crosslinking and subsequently modified with phosphoserine. Briefly, 5 g of polymeric backbone was dissolved in 50 mL of Dulbecco's PBS (DPBS) at 60°C under constant stirring for 30 min. Methacrylic anhydride (16.8 mmol, Sigma Aldrich, 276685) was added gradually at 0.5 mL/min, and the reaction was stirred at 60°C for 2, yielding a degree of methacrylation (DoM) of approximately 70%, as confirmed by ^1^H‐NMR analysis. The solution was then diluted fourfold with DPBS, dialyzed against distilled water at 50°C for 5 days, and freeze‐dried for 4 days. Phosphoserine (Flamma, 17885‐08‐4) was conjugated to the methacrylated gelatin using carbodiimide‐mediated coupling. Briefly, 0.5 g of lyophilized polymer was dissolved in 25 mL of MES buffer at 50°C, and the pH was adjusted to 5. EDC/NHS (3.5 mg.mL^−1^, Thermo Fisher Scientific, 03450 and 24510) activation was conducted under stirring for 15 min at 37°C. Phosphoserine (25 mm) was then added, and the reaction mixture was stirred for 8 h. The mixture was purified by dialysis for 5 days and filtered. Subsequently, it was autoclaved for sterilization and lyophilized for 4 days.

Polymer chemical functionalization was confirmed by ^1^H‐NMR and Fourier‐transform infrared spectroscopy (FTIR). NMR spectra were obtained by using a 400 MHz Bruker Avance NEO spectrometer. Briefly, 18 mg of the modified polymer were dissolved in 500 µL of deuterium oxide (D_2_O) and analyzed at 40°C. An unmodified gelatin sample was analyzed under identical conditions. FTIR spectra were recorded using a Nicolet 6700 FTIR spectrometer (Thermo Fisher Scientific, USA) at room temperature. Representative spectra are provided in the Figure S3a,b).

For fabrication of hydrogel precursors, lyophilized pre‐polymer was dissolved in PBS at polymer contents (PC) of 10, 15, and 20 wt.%. Lithium Phenyl (2,4,6‐trimethylbenzoyl)phosphinate (LAP, Tocris Bioscience, 6146) was added at a final concentration of 0.02% (w/v) as the photoinitiator. Photo‐polymerization was performed using a 405 nm blue light source at an intensity of 10 mW/cm^2^ for 60 s. These conditions were kept constant for all hydrogel formulations and experiments.

### 2.2 Human Chondrocytes Isolation and Expansion

Cartilage specimens were collected from five patients (31‐year‐old male, 15‐year‐old female, 25‐year‐old male, 23‐year‐old female, and 38‐year‐old male) within the framework of a clinical trial. Chondrocytes were derived from articular cartilage fragments. Obtention and use of patient primary cellular materials followed the regulations of the Biobank of the Department of Musculoskeletal Medicine at the CHUV (Lausanne University Hospital, Lausanne, Switzerland) and were registered in the CHUV‐DAL Department Biobank under an ethics protocol approval and following the applicable biobanking directive (BB_029_DAL). Autologous chondrocytes were isolated by enzymatic digestion using sequential pronase and collagenase treatments and then seeded in monolayer culture. Cells were expanded in Dulbecco's Modified Eagle Medium (DMEM) and Ham's F‐12 Nutrient Mix with L‐glutamine (Thermo Fisher Scientific, 11765‐057), supplemented with 10% v/v human platelet lysate (hPL, Sexton Biotechnologies, PL‐SP‐100) and 25 µg/mL L‐ascorbic acid (Sigma Aldrich, A8960).

### 2.3 Cells Encapsulation and Hydrogel Crosslinking

All encapsulation experiments were performed with P2 HACs. For encapsulation, cells were resuspended in hydrogel precursor solutions at a density of 15 × 10^6^ cells/mL, corresponding to clinically relevant concentrations for ACI [14]. The cells were first resuspended in medium and then mixed with a concentrated hydrogel precursor (15, 22.5, and 30 wt.%) at a 1:2 volume ratio. This mixing step adjusted both the polymer content and the cell number. Then suspensions were injected into Teflon cylindrical molds (5 mm diameter × 2 mm height) and photocrosslinked with a light source. The constructs were subsequently transferred into chondrogenic medium consisting of DMEM and Ham's F‐12 supplemented with 10% v/v hPL, 1 wt.% penicillin‐streptomycin, 25 µg/mL L‐ascorbic acid, ITS 1× (Sigma‐Aldrich, I3146), 1.25 mg/mL human serum albumin (CSL Behring AG, 3665734), and 10 ng/mL transforming growth factor‐β1 (TGF‐β1, PeproTech 100–21). The cell‐laden constructs were cultured for 21 days, and the incubation medium was refreshed three times per week. The analyses were performed at weekly timepoints.

### 2.4 Cell Viability and Distribution

Cell viability was assessed at days 1, 7, 14, and 21 using a Live/Dead assay (Biotium, Fremont, CA, USA). Accordingly, cells were stained in PBS containing calcein AM (0.2 µL/mL) for viable cells and ethidium homodimer (0.4 µL/mL) for dead cells. To assess cell distribution in hydrogel cross‐sections, cylindrical constructs were bisected into halves and incubated in Live/Dead solution for 20 min. They were then washed with PBS and imaged using an Evident Fluoview FV4000 confocal laser scanning microscope. Viability was determined by analyzing z‐stack images using FIJI, and live and dead cells were quantified as percentages of the total cells.

### 2.5 Biochemical Assays

Glycosaminoglycan (GAG) production was quantified at weekly time‐points using the dimethylmethylene blue (DMMB) assay. Briefly, the digestion buffer was prepared by dissolving 7.1 g sodium phosphate dibasic (Na_2_HPO_4_), 1.86 g EDTA, and 870 mg L‐cysteine HCl in 500 mL deionized water at pH 6.5. The constructs were enzymatically digested in papain solution containing 6 µL/mL papain (Sigma P‐3125) in digestion buffer at 60°C overnight. For each measurement, 20 µL of the digested sample was loaded into a 96‐well plate with 130 µL of DMMB dye in triplicate, and absorbance at 590 nm was read with a Wallac 1420 Victor2 microplate reader (PerkinElmer, Ramsey, MN, USA). GAG content was normalized to DNA content determined by Hoechst 33,258 fluorescence assay (Thermo Fisher Scientific). The emission at a wavelength of 460 nm was measured, and the DNA concentrations were interpolated using a standard curve based on chondroitin sulphate (Sigma Aldrich, C6737). Results were reported as GAG/DNA (µg/µg).

### 2.6 Histological Analysis

For histology, cell‐laden hydrogels were fixed in 10% formalin overnight, dehydrated through a graded ethanol series up to 100%, and treated in xylene. The samples were then embedded in paraffin and sectioned at 5 µm thickness using a microtome. Sections were stained with hematoxylin and eosin (H&E) for general morphology, Alcian Blue (AB) for sulfated glycosaminoglycans, and Safranin O (Saf‐O) for proteoglycan distribution, and the imaging was performed using a VS200 whole slide scanner.

### 2.7 Immunohistochemistry

Immunohistological analysis was performed for the evaluation of collagen type II and aggrecan deposition. Briefly, the prepared sections were deparaffinized and rehydrated. Antigen retrieval was carried out by incubation with proteinase K (20 µg/mL in TE buffer, pH 8.0) for 15 min at 37°C. Endogenous peroxidase activity was quenched using BLOXALL solution, followed by blocking with 2.5% normal horse serum for 2 h. Subsequently, we incubated the sections overnight at 4°C with primary antibodies against collagen II at a dilution of 1:500 (Invitrogen, MA5‐12789) and aggrecan at a dilution of 1:1000 (Invitrogen, AHP0022). Detection was then performed using the ImmPRESS HRP horse anti‐mouse IgG polymer reagent (secondary antibody system) for 2 h, and staining was visualized with 3,3′‐diaminobenzidine (DAB) using the ImmPACT DAB substrate kit.

### 2.8 Mechanical Testing

Unconfined compression testing was performed to evaluate the mechanical properties of the constructs at a weekly interval using an Instron E3000 (Norwood, MA, USA). The samples were compressed at a strain rate of 0.1 mm × s^−1,^ and the compressive modulus was calculated from the slope of the true stress–strain curve (n = 3). Acellular hydrogels were also prepared and tested under identical conditions as cell‐laden constructs to assess intrinsic hydrogel stability.

### 2.9 Statistical Analysis

All data are reported as mean ± standard deviation (SD). Data were primarily analyzed using a linear mixed‐effects model (LMM) with polymer content and timepoint as fixed effects and donor as a random effect to account for repeated measures and inter‐donor variability. Two‐way repeated‐measures ANOVA with the Geisser–Greenhouse correction was also used to analyze main and interaction effects, followed by Tukey's post‐hoc pairwise comparisons.

## 3 Results

Chondrocytes encapsulated within the adhesive hydrogel maintained high viability across all formulations and timepoints, although significant effects of polymer content and culture duration were observed. Live/Dead staining demonstrated that cell viability exceeded 85% at day 1 in all hydrogels after encapsulation and remained above 70% at day 21. Even in the highest polymer content formulation (20%), viability remained consistently above 70% across all timepoints. Quantitative analysis revealed significant main effects of hydrogel PC (*p* < 0.001) and time point (*p* < 0.001) on viability, with a significant interaction between the two factors. The LMM further indicated low variability attributable to individual donors (Group Var coefficient = 0.60), whereas donor effects were not significant in the ANOVA (p = 0.815), consistent with the within‐subject design. Moreover, there is no significant change in variability over time. Hydrogel comparisons demonstrated that 20 wt.% hydrogels supported significantly lower viability than 10 wt.% hydrogels when averaged across time (*p* < 0.001). In contrast, no statistically significant differences were found between 10 wt.% and 15 wt.% PC (p = 0.1664). Across all hydrogel formulations, viability decreased from day 0 to day 21, there was no significant difference between days 14 and 21 for 10 wt.% (p = 0.9809) and 15 wt.% PC (p = 0.0702) hydrogels.

**FIGURE 1:**
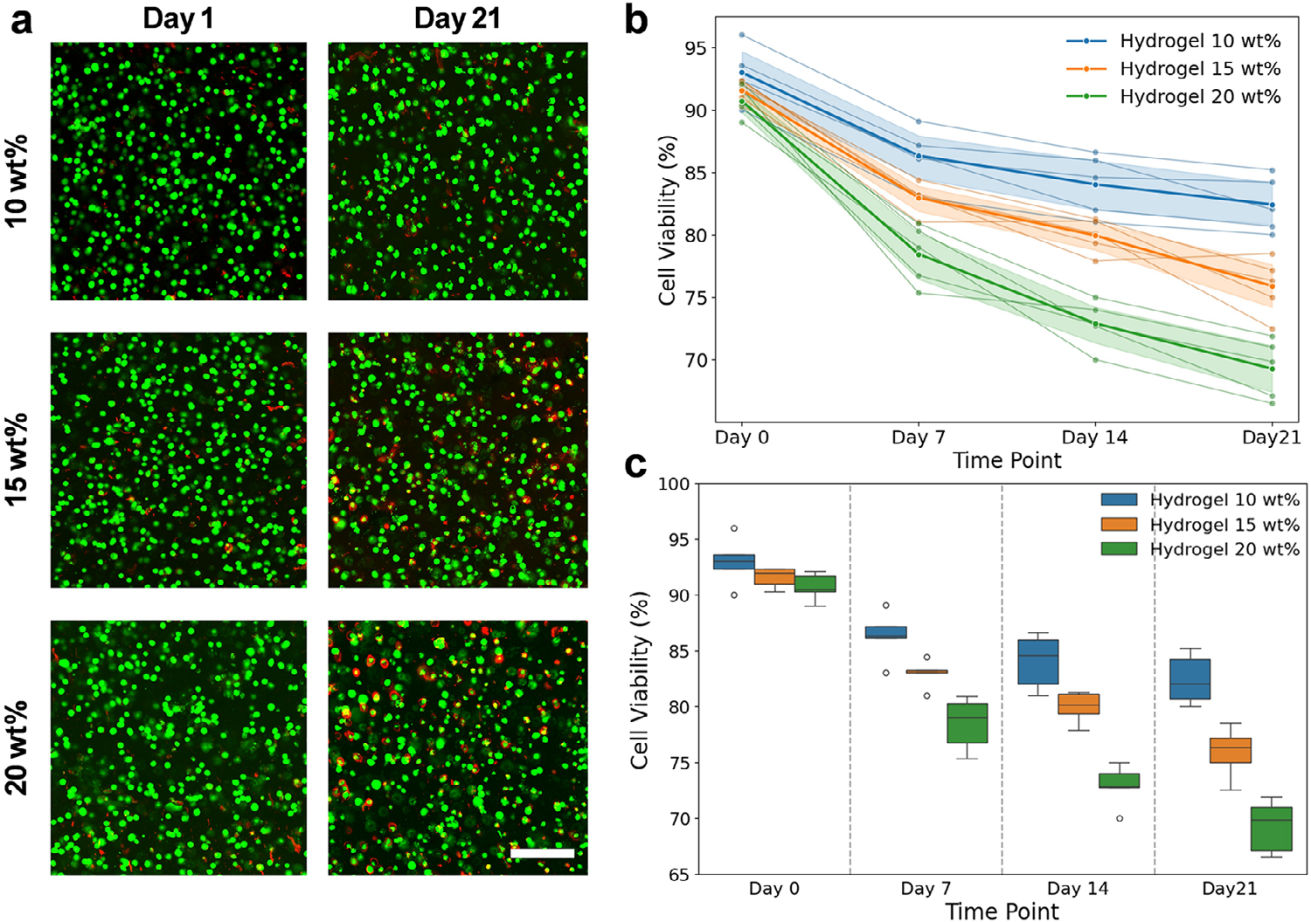
Cell viability and distribution within hydrogels. (a) Representative confocal Live/Dead images of encapsulated human chondrocytes in 10, 15, and 20 wt.% hydrogels at days 1 and 21 (38‐year‐old male donor). Live cells are imaged using the green stain. Samples are cut into two halves, and images are taken from the cross‐section. Scale bar = 150 µm. (b) Quantification of viability and overall trend for each hydrogel and variability in response among 5 HAC donors is shown by a spaghetti plot. The thinner lines represent the individual viability for each cell donor, and the thicker lines represent the mean ratio with shaded areas (95% confidence interval) for each polymer content. (c) Box plot showing the distribution of viability across all donors. (n = 3).

Confocal microscopy confirmed a uniform 3D distribution of cells within the hydrogel constructs. No aggregation or clustering of HACs was observed, indicating a homogeneous encapsulation environment and sufficient precursor viscosity during encapsulation to prevent sedimentation prior to crosslinking (Figure 1).

### 3.1 Extracellular Matrix Production

Biochemical analysis showed progressive deposition of glycosaminoglycans (GAGs) normalized to DNA content for all hydrogels over the culture period. By day 21, the 10 wt.% and 15 wt.% hydrogels exhibited significant gains relative to day 7 (*p* < 0.001), with around a four‐fold increase in the 10 wt.% formulation. The 20 wt.% hydrogel also accumulated marked GAG, although to a lesser extent, with an approximately two‐fold increase (p = 0.0031).

**FIGURE 2:**
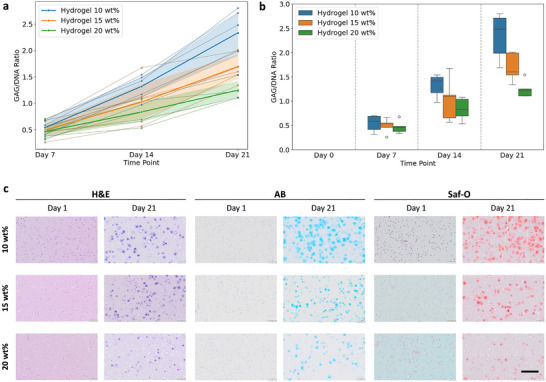
Glycosaminoglycan accumulation and histological staining. (a) Overall trend of GAG/DNA accumulation for each hydrogel and variability in response among 5 HAC donors shown by spaghetti plot. The thinner lines represent the individual GAG/DNA ratio for each cell donor, and the thicker lines represent the mean ratio with shaded areas (95% confidence interval) for each polymer content. (b) Box plot showing the distribution of the GAG/DNA ratio. (c) Representative histological sections at day 1 and 21 (25‐year‐old male donor), stained with H&E, Alcian Blue (AB), and Safranin O (Saf‐O). Scale bar = 200 µm.

In addition to significant effects of hydrogel formulation (*p* < 0.001), we observed a significant interaction between the hydrogel type and time point effects (*p* < 0.001), which indicates specific trends of GAG accumulation, depending on polymer content. Overall, stiffer hydrogels with high PC supported less GAG accumulation. Unlike viability outcomes, donor effects reached statistical significance in the ANOVA (*p* < 0.001). Similarly, the LMM findings demonstrated considerable variability attributable to donor effects. This indicates that autologous chondrocytes from each patient play a distinct role in extracellular matrix formation capacity (Figure 2a,b). Variability increased over the 3‐week culture time, mainly reflecting donor‐specific differences in matrix production. This effect was more pronounced in lower polymer content formulations that supported higher overall matrix synthesis and therefore amplified donor‐related differences.

### 3.2 Histological and Immunohistological Analysis

Histological assessment of extracellular matrix organization further supported our quantitative findings. Consistent with our confocal viability imaging, H&E staining revealed homogeneous cell distribution in hydrogel networks for all formulations, with cells retaining rounded morphologies. Unlike 2D membrane‐based seeding systems, where chondrocytes tend to dedifferentiate and lose their capacity for active ECM deposition [15, 16], this spherical phenotype is consistent with a chondrogenic lineage and indicates that the hydrogel microenvironment allows for cartilage‐specific matrix synthesis (Figure 2c and Figure S1a).

Alcian Blue staining demonstrates progressive deposition of sulfated GAGs around the chondrocytes. The staining was more intense in the lower PC formulation by day 21, with uniform distribution throughout the constructs. Safranin O staining is in line with these observations and shows considerable proteoglycan deposition. According to the pattern of staining in the sections, matrix production is not confined to the periphery of the constructs but extended throughout the hydrogel volume, unlike the predominantly surface deposition effect seen in membrane‐based ACI (Figure 2c and Figure S1b,c).

**FIGURE 3:**
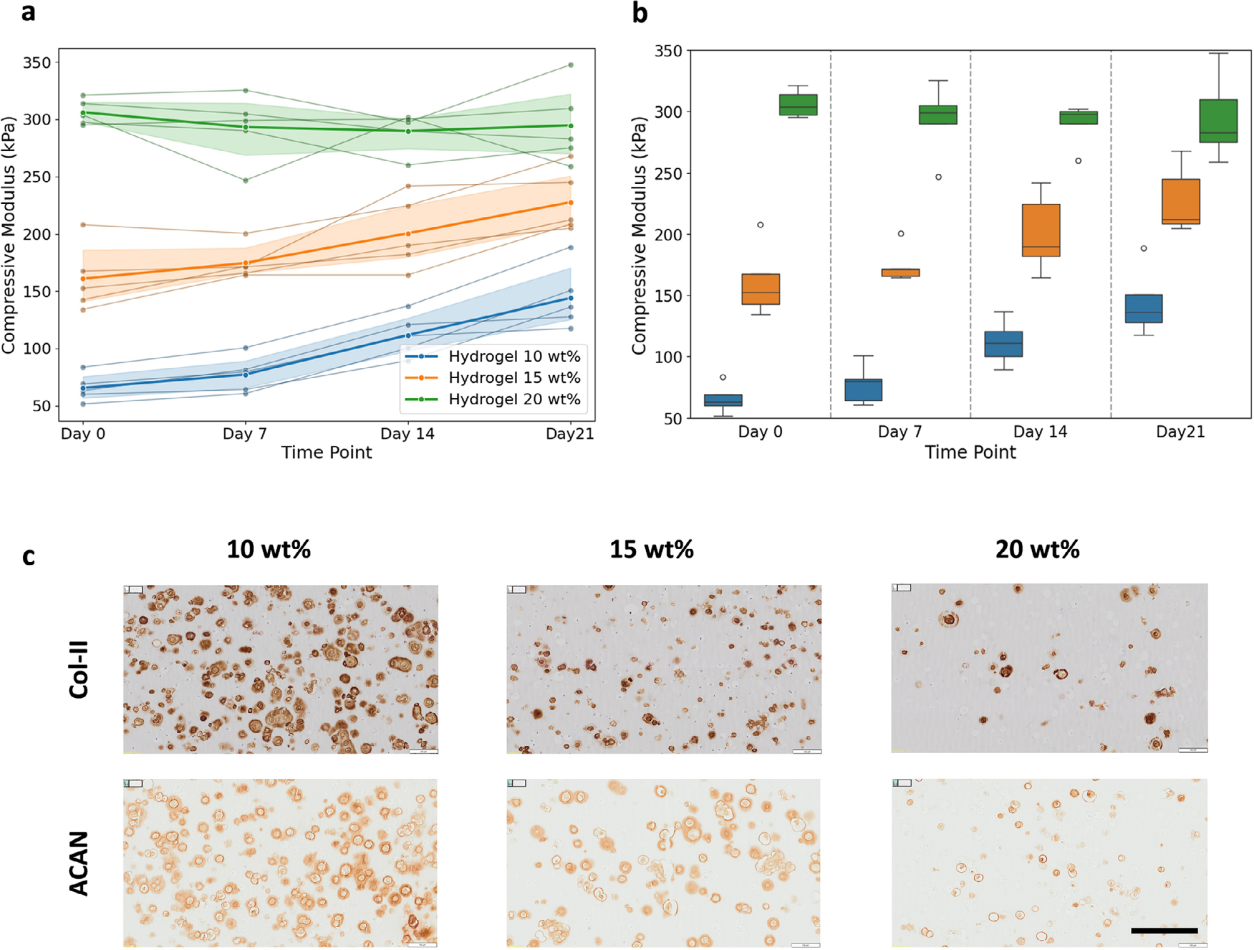
Compressive modulus measured at different time points. (a) Spaghetti plot showing donor‐specific trajectories and variability, and (b) box plot summarizing the overall distribution of compressive modulus data across donors. (c) Representative immunohistological sections at day 21 (38‐year‐old male donor), stained with H&E, Alcian Blue (AB), and Safranin O (Saf‐O). Scale bar = 200 µm. Increasing compressive modulus corresponds to progressive expression of aggrecan and collagen II, indicating functional maturation.

Collagen II, the principal fibrillar collagen of hyaline cartilage, was expressed in all formulations by day 21. Staining was distributed uniformly and reflected active synthesis of cartilage‐specific proteins. In particular, the 10% and 15% PC groups demonstrated the most intense staining, aligning with the biochemical data. Aggrecan, as the major proteoglycan responsible for cartilage compressive resilience, exhibited a staining pattern parallel to that of collagen II. This confirms that chondrocytes remained functionally active in synthesizing key structural components of hyaline cartilage. However, the 20% hydrogel formulation displayed weaker staining intensity with more patchy distribution, consistent with its lower biochemical GAG accumulation (Figure 3c and Figure S2a,b). The histological findings confirm the quantitative biochemical results on matrix composition and accumulation, and the immunohistochemical analysis provides evidence of functional maturation and matrix organization closely associated with mechanical performance.

### 3.3 Mechanical Performance

Unconfined compression testing showed progressive increases in the mechanical performance of the hydrogel constructs, consistent with progressive ECM deposition. Statistical analyses demonstrated significant main effects of hydrogel formulation (*p* < 0.001) and its significant interaction with culture time (*p* < 0.001), indicating that the evolution trend of compressive modulus over time depends on polymer content. Similar to the GAG accumulation outcome, donor variability contributed significantly to mechanical outcomes. In ANOVA, donor effects were significant (*p* < 0.001), while the LMM with random slopes confirmed substantial variability attributable to individual donors. This indicates that although our hydrogel carrier maintains reproducible viability of encapsulated cells and provides functional enhancement, the biochemical and mechanical performance are further influenced by the quality of patient cells (Figure 3a,b).

The 10 wt.% formulation exhibited the lowest initial modulus but showed the most pronounced relative increase during culture (p = 0.0002), whereas the 15 wt.% hydrogel demonstrated intermediate initial stiffness with significant reinforcement over time (p = 0.0026). The 20 wt.% formulation maintained the highest modulus at all timepoints, but its relative increase was negligible and did not reach statistical significance compared to day 1 (p = 0.9935). However, it is expected that mechanical enhancement would be observed over longer culture times, as all formulations provided progressive matrix deposition. It should be noted that this behavior was recorded over 3 weeks, and longer timepoints could give further insights. While stiffer hydrogels provide immediate mechanical stability, softer formulations allow for greater reinforcement by matrix deposition during culture. The 15 wt.% formulation emerged as an optimal compromise combining sufficient stiffness with meaningful gains in modulus over time.

To distinguish the contribution of newly synthesized extracellular matrix from intrinsic hydrogel changes over time, acellular hydrogels were cultured under identical conditions and mechanically tested at day 0 and day 21. The compressive modulus of acellular hydrogels showed even a minor decrease, particularly in lower PC formulations (Table S1). These findings indicate that the progressive increase in compressive modulus observed in cell‐laden constructs is primarily attributable to extracellular matrix deposition.

## 4 Discussion

Cartilage cellular therapies have evolved considerably since their initial clinical application. However, effective cell delivery and maintenance of a supportive environment for cellular function are major challenges in the field. Current delivery systems in advanced ACI generations (e.g., MACI) and other cell‐based strategies often fail to secure cells at the defect site or to provide an environment conducive to matrix production [11, 17, 18]. Consequently, the next generation of cell carriers should not only overcome the limitations of existing therapeutic delivery approaches but also actively support appropriate cellular behavior. Despite advances in hydrogel‐based carriers, their application has not resulted in meaningful progress in the clinical outcomes of cartilage therapies [11]. One of the clinically available injectable examples is Novocart Inject, which is a hyaluronan‐based biomaterial currently in a Phase III clinical trial in Germany [9, 19]. While this system has shown early promise, it exhibits several limitations, such as relatively weak initial bonding strength [10] and incomplete integration in a significant proportion of patients as observed by MRI at 12 months after surgery [20].

The adhesive hydrogel presented in this study showed potential for addressing these challenges. Following its successful demonstration of tissue integration in cartilage repair in a large animal model, it also showed strong potential for autologous chondrocyte delivery, as evidenced by encapsulation analyses using cells from different donors. The hydrogel allows for uniform 3D encapsulation of HACs at clinically relevant densities and creates a microenvironment that maintains acceptable viability and promotes matrix production and mechanical reinforcement over time. Indeed, while the superior adhesion to cartilage tissue has been confirmed previously in an acellular setting by in vitro and in vivo characterization, here we show that this system maintains this advantage while supporting cell encapsulation and matrix deposition. Future studies will evaluate adhesion performance directly in the presence of encapsulated cells and during tissue maturation. Compared to MACI, for instance, our hydrogel could significantly simplify the clinical workflow by avoiding membrane seeding and overcoming the 2D seeding approach, as well as the potential to eliminate the fixation problem with the adhesive property. Its injectability and fast light‐curable stabilization are compatible with minimally invasive arthroscopic delivery and potentially expand access to ACI beyond specialized centers. However, it is important to note that the present findings are based on in vitro evaluation, and further studies will be required to confirm integration and long‐term functionality.

By systematically comparing polymer content formulations for HACs encapsulation, we could optimize the hydrogel in the context of biomechanical and biological outcomes and identified the 15 wt.% PC hydrogel as the optimal balance between initial stability and stiffness, cell viability, and long‐term tissue formation for future translational studies. The 10 wt.% hydrogel supported the highest relative gains in matrix production and compressive modulus, but at the expense of low initial mechanical stability. On the other hand, the 20 wt.% hydrogel provided high baseline stiffness but slower ECM accumulation. The 15 wt.% formulation offered the best compromise: sufficient initial mechanical integrity for defect stabilization combined with progressive reinforcement through matrix synthesis and viability. This tunable balance between mechanics and biology is a critical feature for clinical translation, where both immediate fixation and long‐term tissue integration are required.

While viability decreased modestly over the culture time, it remained above 70% even in the stiffest hydrogels, which is in the recommended viability range [21, 22]. Importantly, the remaining viable cells were functionally active, as evidenced by GAG accumulation, collagen II and aggrecan synthesis, and mechanical enhancement. Although further reductions in viability after three weeks cannot be excluded, the observed plateau between days 14 and 21 in the 10 wt.% and 15 wt.% hydrogels suggests stabilization rather than progressive decline. In vivo studies will be required to confirm this evolutionary behavior.

Notably, we observed variability among human donors in terms of matrix production by HACs and mechanical outcomes of the constructs, which indicates the influence of patient‐specific cellular capacity in autologous applications. This is in line with the clinical experience of ACI, where patient outcomes vary despite standardized procedures [1]. Importantly, our hydrogel system consistently supported cell encapsulation, viability, and baseline function across all donors, which suggests it can accommodate this biological diversity.

### 4.1 Limitations and Future Perspectives

The present study was limited to the in vitro culture of autologous chondrocytes over three weeks. While early matrix production can be analyzed in this timeframe, longer‐term studies are required to assess sustained viability, hydrogel degradation, and maturation of the newly formed tissue. While the adhesive hydrogel has been previously shown to achieve strong tissue integration in an acellular setting, the present study did not directly assess adhesion in the presence of encapsulated cells. Therefore, the impact of cell encapsulation on adhesion strength and long‐term mechanical integrity remains to be investigated in our future studies. In addition, in vivo models will be essential to evaluate the long‐term functional outcomes, as planned for our future investigations. Finally, while this study focused on HACs, it will be important to assess the versatility of the system for other clinically relevant cell sources, including allogeneic cells, which may offer logistical advantages. Despite these limitations, the obtained results have important translational implications.

## 5 Conclusion

In this study, we investigated an adhesive light‐activated hydrogel as a promising platform for next‐generation autologous chondrocyte implantation. With a supportive 3D environment for chondrocyte function, this system can bridge the gap between injectable cell carriers and cartilage integration and healing. Through systematic optimization of mechanical and biological performance, we identified a hydrogel formulation (15 wt.%) that achieves an optimal balance between initial stability and regenerative capacity. Importantly, this study demonstrates the potential for functional chondrocyte support to be achieved within a single injectable system, a combination that remains a long‐standing limitation of current cellular therapies. While the adhesive capabilities have been established in prior studies, they will be further evaluated in the cell‐laden condition. The findings presented here form the basis for our subsequent investigation of adhesion performance in cell‐laden hydrogels and support future in vivo validation of this hydrogel system for enhanced therapeutic delivery in regenerative orthopaedics.

## Conflicts of Interest

The authors declare no conflict of interest.

## Supporting information




**Supporting File**: mabi70178‐sup‐0001‐SuppMat.docx.

## Data Availability

The data that support the findings of this study are available from the corresponding author upon reasonable request.
