# Supplementary material for: In Vitro Evaluation of an Adhesive Hydrogel‐Based Chondrocyte Carrier for Enhanced Therapeutic Delivery
Source: Macromol Biosci. 2026 Apr 9;26:e00653. doi: 10.1002/mabi.202500653 (PMC13064798; doi:10.1002/mabi.202500653)
Supplement: Supplementary file 1 — Supporting File: mabi70178‐sup‐0001‐SuppMat.docx. [file MABI-26-e00653-s001.docx]

**Supporting Information**

**In vitro evaluation of an adhesive hydrogel-based chondrocyte carrier for enhanced therapeutic delivery**

Peyman Karami ^1,2 #^, Alexis Laurent ^3,4^, Virginie Philippe ^1,4^, Lee Ann Applegate ^4,5,6^, Dominique P. Pioletti ^2^, Robin Martin ^1* #^

^1^ Department of Orthopedic Surgery and Traumatology, Lausanne University Hospital, University of Lausanne, CH-1011 Lausanne, Switzerland

^2^ Laboratory of Biomechanical Orthopaedics, Institute of Bioengineering, School of Engineering, EPFL, CH-1015 Lausanne, Switzerland

^3^ Manufacturing Department, LAM Biotechnologies SA, CH-1066 Epalinges, Switzerland

^4^ Regenerative Therapy Unit, Reconstructive and Hand Surgery Service, Lausanne University Hospital, University of Lausanne, CH-1066 Epalinges, Switzerland

^5^ Center for Applied Biotechnology and Molecular Medicine, University of Zurich, CH-8057 Zurich, Switzerland

^6^ Oxford OSCAR Suzhou Center, Oxford University, Suzhou 215123, China

^*^ corresponding author. Email: robin.martin@chuv.ch

**a**


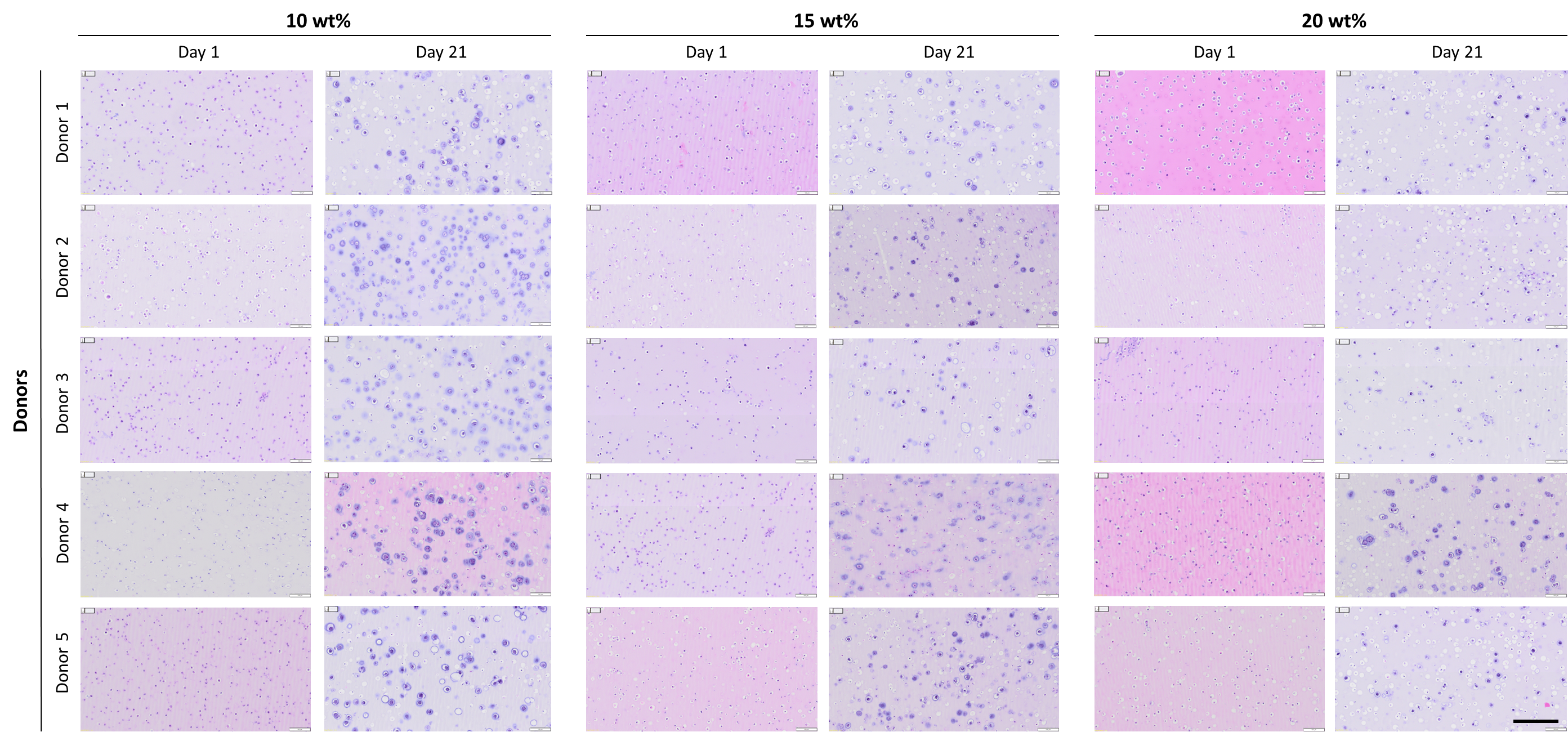


**b**


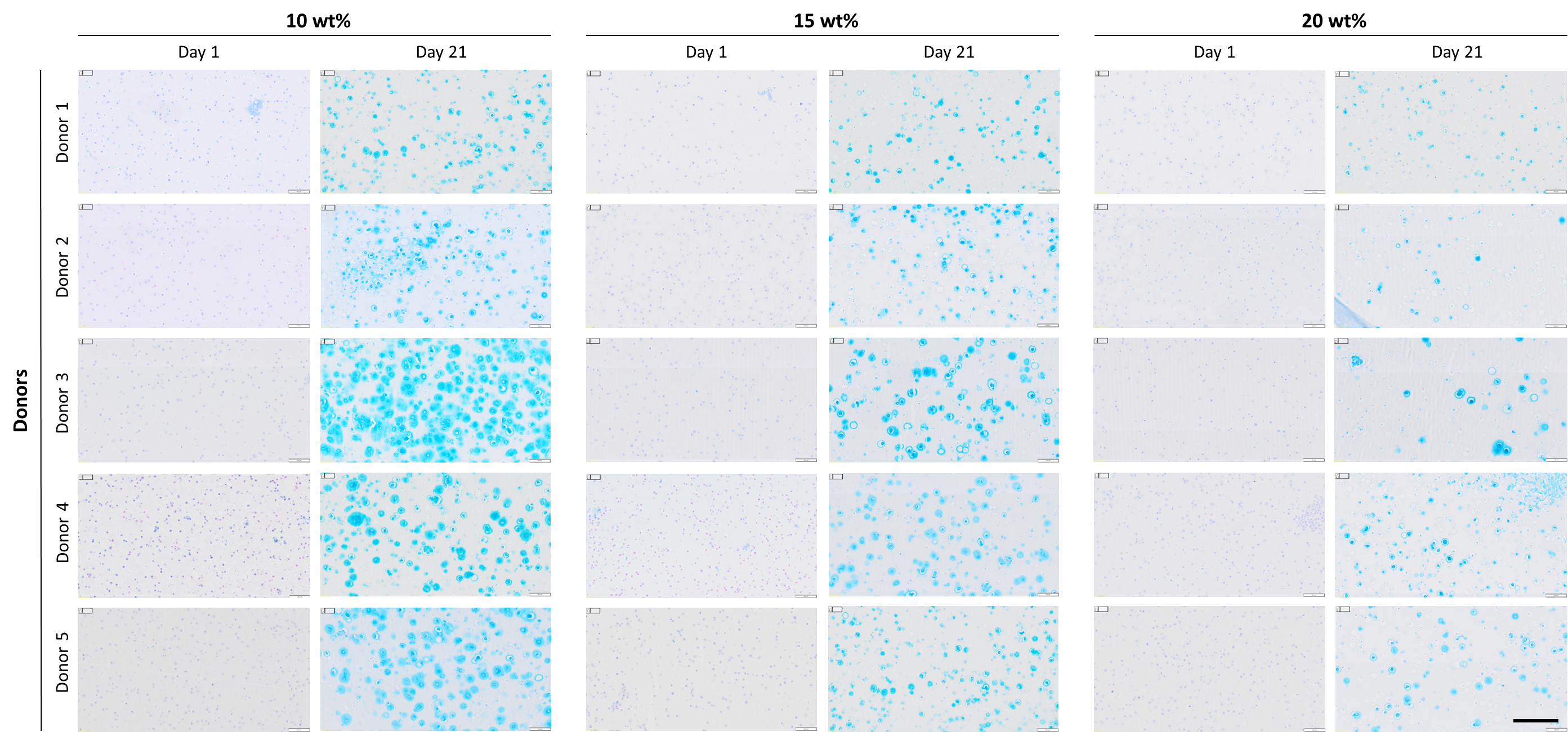


**c**


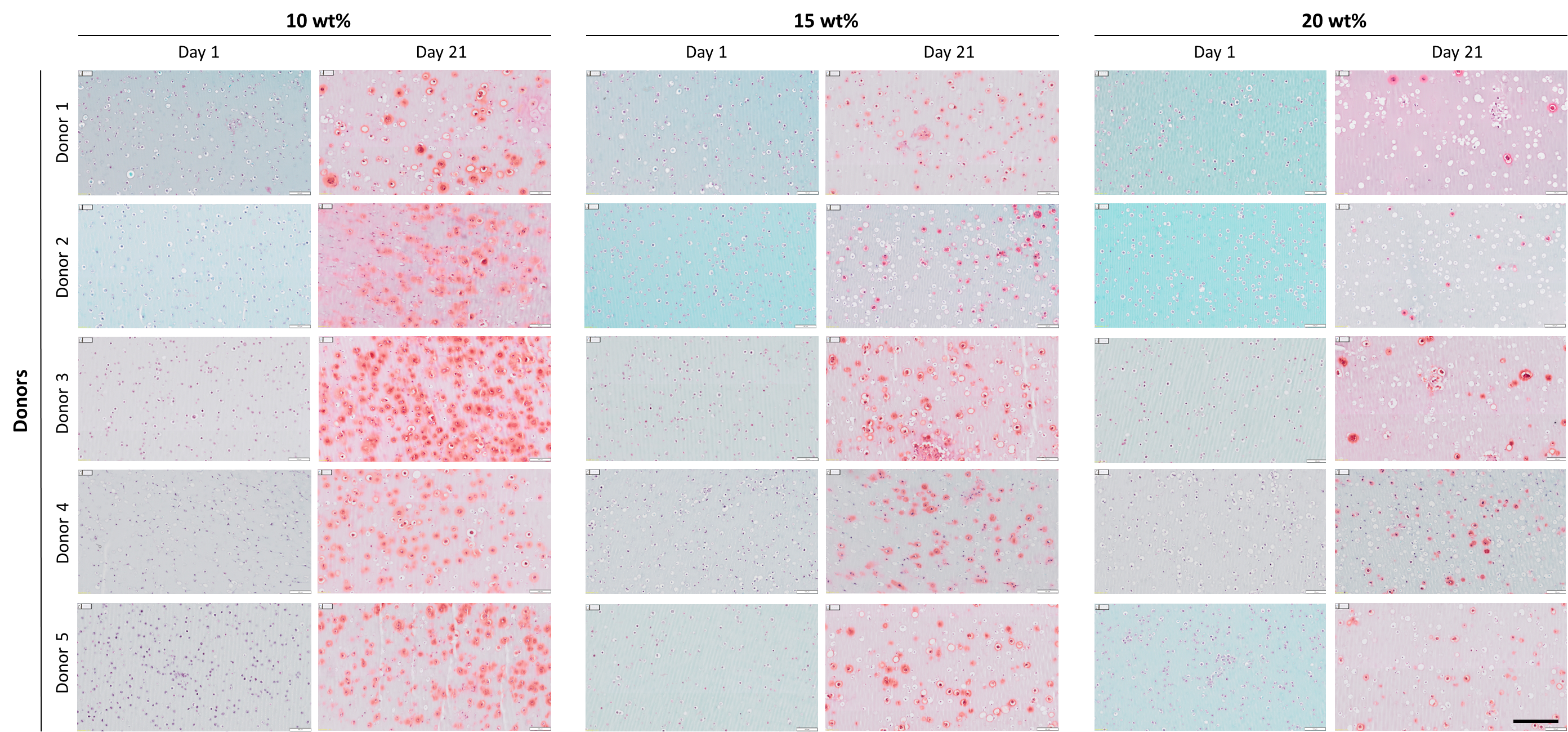


**Supplementary Figure 1.** Representative histological sections from all five donors stained with (a) hematoxylin and eosin (H&E), (b) Alcian Blue (AB) and (c) Safranin O (Saf-O) at day1 and 21. Rows correspond to donors and columns show hydrogel formulations (10%, 15%, and 20%). Scale bar = 200 μm.


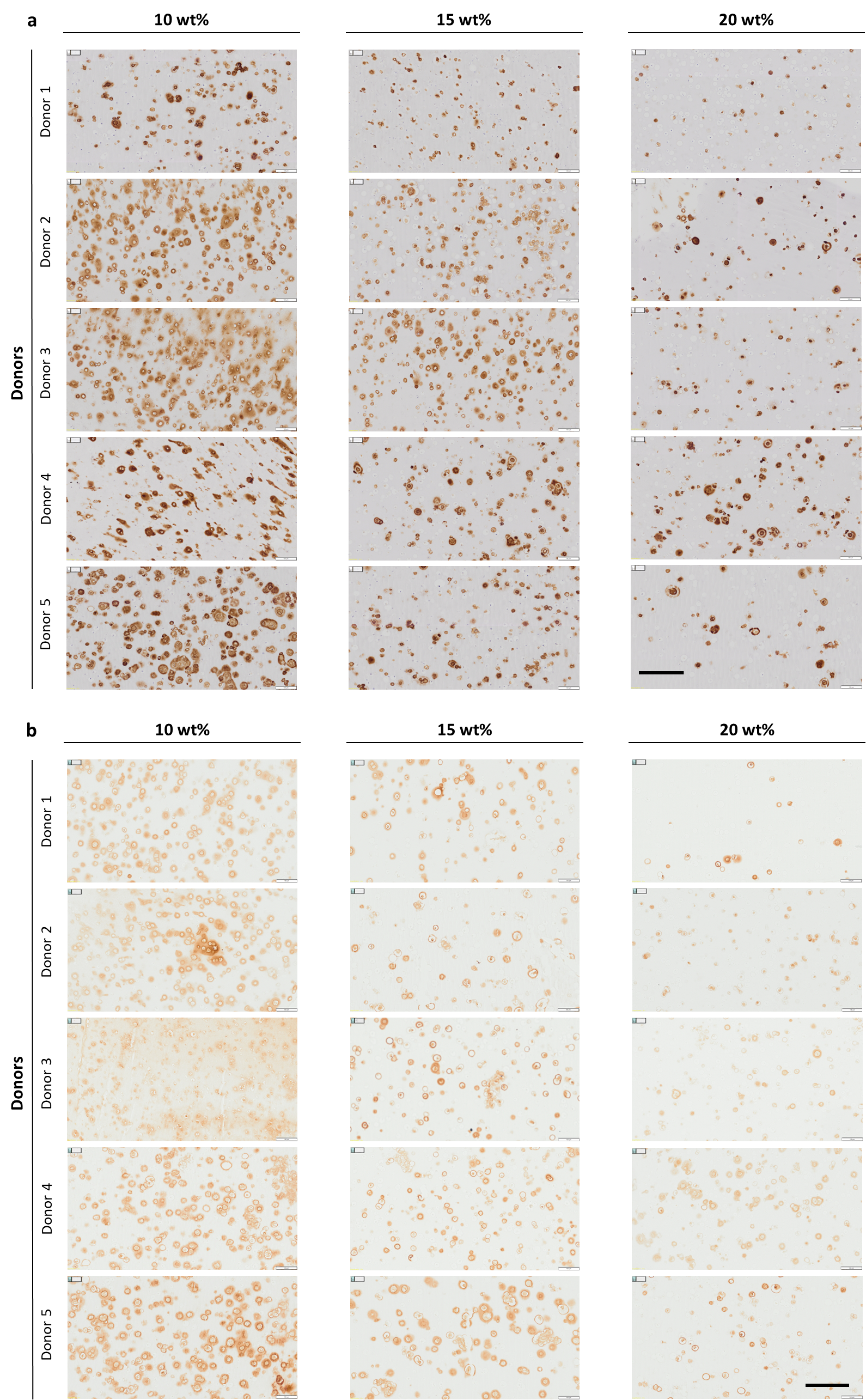


**Supplementary Figure 2.** Immunohistochemical staining for collagen II (top) and aggrecan (bottom) in constructs from all five donors cultured in 10%, 15%, and 20% hydrogels at day 21. Scale bar = 200 μm.

**
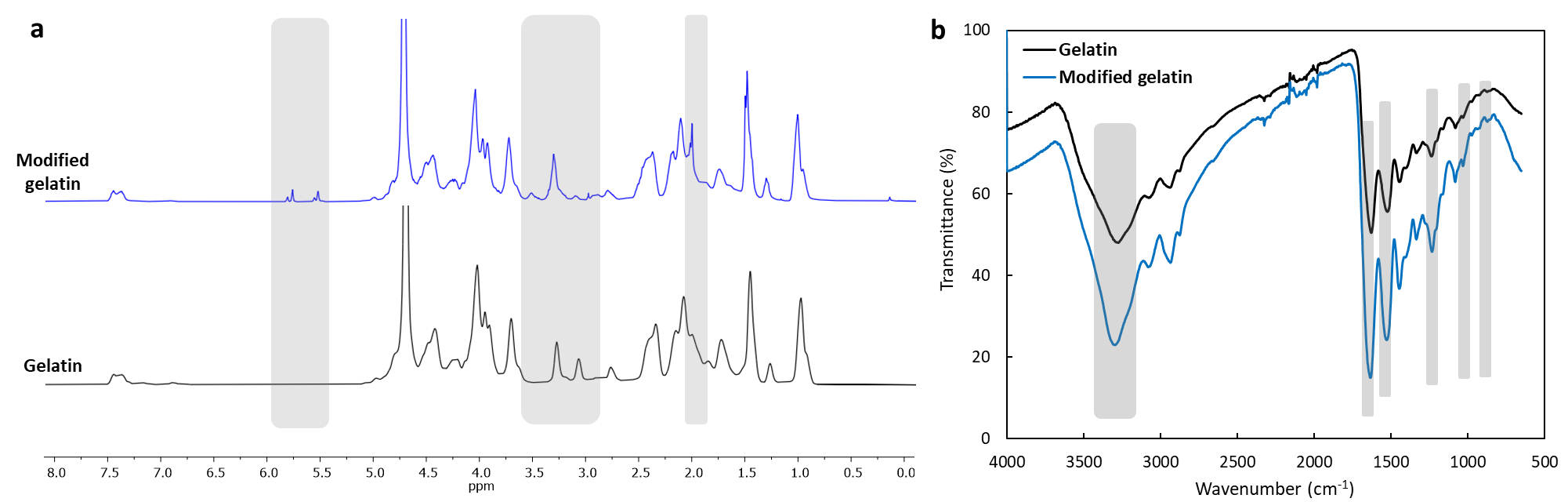
**

**Supplementary Figure 3. (a)** Representative ¹H-NMR spectrum of modified polymeric backbone confirming methacrylation of gelatin, showing characteristic peaks corresponding to methacrylate vinyl protons. **(b)** FTIR spectra of modified gelatin showing characteristic peaks corresponding to functional groups. The spectra show increased intensity of the characteristic amide bands at ~1630 cm⁻¹ (amide I), ~1546 cm⁻¹ (amide II), and ~1244 cm⁻¹ (amide III), along with a broad band at 3200–3400 cm⁻¹ assigned to N–H and O–H stretching, indicating modification of the gelatin structure. Additionally, the modified sample exhibits increased absorbance in the 920–1080 cm⁻¹ region, attributed to P–O stretching vibrations.

**Supplementary Table 1.** Compressive modulus of acellular hydrogels cultured under identical conditions.

| **Polymer content** | **Day 0 (kPa)** | **Day 21 (kPa)** |
| --- | --- | --- |
| 10 wt% | 117.0 ± 25.4 | 102.3 ± 18.2 |
| 15 wt% | 320.6 ± 32.6 | 308.7 ± 29.9 |
| 20 wt% | 456.8 ± 31.5 | 451.1 ± 33.7 |
